# SnSe-Coated Microfiber Resonator for All-Optical Modulation

**DOI:** 10.3390/nano12040694

**Published:** 2022-02-19

**Authors:** Lei Chen, Jingyuan Ming, Zhishen Zhang, Jumei Shang, Lingyun Yu, Heyuan Guan, Weina Zhang, Zefeng Xu, Wentao Qiu, Zhe Chen, Huihui Lu

**Affiliations:** 1 Guangdong Provincial Key Laboratory of Optical Fiber Sensing and Communications, Department of Optoelectronic Engineering, Jinan University, Guangzhou 510632, China; lynnchan538@stu2018.jnu.edu.cn (L.C.); mingjingyuan@outlook.com (J.M.); 1934021043@stu2019.jnu.edu.cn (J.S.); llingyunyu@163.com (L.Y.); vina_zhang001218@163.com (W.Z.); xuzefeng134210@163.com (Z.X.); 2 School of Physics and Optoelectronic Technology, South China University of Technology, Guangzhou 510641, China; zhishenzhang@yeah.net; 3 Key Laboratory of Optoelectronic Information and Sensing Technologies of Guangdong Higher Education Institutes, Department of Optoelectronic Engineering, Jinan University, Guangzhou 510632, China; thzhechen@163.com (Z.C.); thuihuilu@jnu.edu.cn (H.L.)

**Keywords:** microfiber-knot resonator, tin selenide, all optical modulation, resonance enhance

## Abstract

In this study, a tin monoselenide (SnSe)-based all-optical modulator is firstly demonstrated with high tuning efficiency, broad bandwidth, and fast response time. The SnSe nanoplates are deposited in the microfiber knot resonator (MKR) on MgF_2_ substrate and change its transmission spectra by the external laser irradiation. The SnSe nanoplates and the microfiber are fabricated using the liquid-phase exfoliation method and the heat-flame taper-drawing method, respectively. Due to the strong absorption and enhanced light–matter interaction of the SnSe nanoplates, the largest transmitted power tunability is approximately 0.29 dB/mW with the response time of less than 2 ms. The broad tuning bandwidth is confirmed by four external pump lights ranging from ultraviolet to near-infrared. The proposed SnSe-coated microfiber resonator holds promising potential for wide application in the fields of all-optical tuning and fiber sensors.

## 1. Introduction

Two-dimensional materials (TDMs) with the tunable optical properties and atomically thin thickness have been excellent candidates for constructing the nanoscale optical modulator [1]. Beside the external electric field and chemical doping, the optical properties of TDMs can be modified by external light excitation, which offers an attractive approach for all optical modulation [2,3]. Compared with the all-optical modulator based on the nonlinear optical effects, the TDM approach has the advantages of large modulation depth, high tuning efficiency, broad bandwidth, and low operating power [4,5]. A series of 2D materials, such as graphene [6], transition metal sulfides [7,8,9], MXenes [10], and black phosphorus [11], have been prepared and integrated into the miniaturized all-optical modulator. The strong light–graphene interaction results in high-speed broadband all-optical modulation with maximum modulation depths of 5 and 13 dB for single- and bi-layer graphene, respectively [12]. In order to be compatible with the optical fiber communication network, TDMs have been integrated on the end face of the fiber, the side of the D-fiber, and the side of the microfiber [13]. Through the evanescent fields, the properties of modes in fiber are regulated with the refractive-index change of TDMs under the external light excitation [14]. An all-optical switch based on a graphene-coated fiber Mach–Zehnder interferometer is reported with mW switching powers [15]. Thanks to the higher energy density and longer interaction distance in the resonator, high sensitivity (0.4 dB/mW) is achieved by a tungsten disulfide (WS_2_)-nanosheet-coated microfiber knot [16].

Among various TDMs, tin monoselenide (SnSe) possesses the exotic optical properties for realizing the all-optical modulator with high performances. The 2D nanosheets of SnSe has the high absorption coefficient up to 7 × 10^4^ cm^−1^ and a broadband photoresponse from ultraviolet to near-infrared (370–808 nm) [17]. SnSe photodetectors are reported with the strong response behavior to a mid-infrared of 10.6 μm [18] and showing an ultrahigh responsivity of 277.3 A/W with a detectivity of 7.6 × 10^11^ Jones [19]. Therefore, the SnSe nanosheet is a competitive candidate for the all-optical control photonics. However, this is rarely reported.

In this study, an all-optical SnSe-coated MKR modulator is experimentally demonstrated. The SnSe nanoplates are deposited around the microfiber (MF) for realizing a tunable hybrid waveguide. Under the external laser irradiation, the largest transmitted power tunability is approximately 0.29 dB/mW with the response time of less than 2 ms. The broad tuning bandwidth is confirmed by four external pump lights ranging from ultraviolet to near-infrared. We present the device fabrication, theoretical analysis, and experimental results of the all-optical control of light functionality and conclusions in subsequent sections.

## 2. Device Fabrication

**Figure 1:**
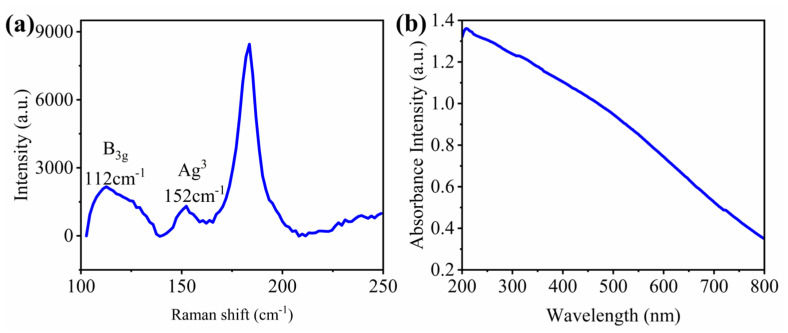
(**a**) Raman spectrum of the SnSe nanosheets. (**b**) Absorption spectrum of the SnSe nanosheets.

The SnSe nanoplates were fabricated by the liquid-phase exfoliation method [20], which involved SnSe single crystal flakes being added to dispersant in reagent bottles using the bath sonication process as dispersion, which was centrifuged in the end. Additionally, the SnSe nanoplates were 1 mg/mL from MUKE NANO Technology Co., Ltd. The finite lateral sizes of SnSe nanoplates are approximately 0.05–1 μm, whereas the thickness varied from 1 to 10 layers. Figure 1a shows the Raman absorption spectrum of the SnSe nanoplates. Two prominent peaks at 112 and 152 cm^−1^ agree well with the B_3g_ and Ag^3^ modes of single-phase SnSe [20,21]. The absorption spectrum (measured by a UV-VIS spectrophotometer) of SnSe nanoplates is shown in Figure 1b. In the wavelength range of 200–800 nm, the SnSe nanoplates keep the strong absorption, which indicates that SnSe-based modulators can be efficiently carried out at wide spectral bands.

**Figure 2:**
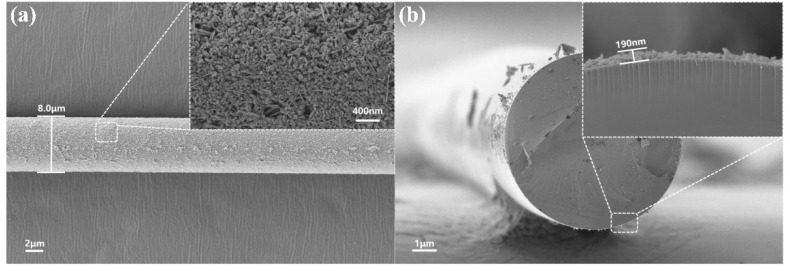
(**a**) SEM images of MF coated with SnSe nanosheets and an enlarged view of the region marked by a dotted rectangle. (**b**) SEM images of the cross-section view of MF coated with SnSe and an enlarged view of the region marked by a dotted rectangle.

The microfiber is fabricated from a single-mode fiber (SMF-28e from Corning Inc.) via taper drawing with a high temperature flame, which is known as the heat-flame taper-drawing method [22]. The diameter of MF for coating the SnSe nanoplate was chosen to be between 6 and 8 μm, which balances the light–matter interaction, insertion loss, and mechanical strength. After an ultrasonic treatment of the SnSe nanoplate (approximately 30 min at a temperature of 25 °C), the oscillating SnSe dispersion is transferred to the microfiber using a needle tube. Then, it takes about for approximately 6 h to allow the solvent to dry naturally. Figure 2a shows that SnSe nanoplates precipitated around the MF with a diameter of 7.1 μm. The inset is an enlarged image of SnSe on the MF from which the closely self-packed SnSe nanoplates are visible. Figure 2b shows a cross-sectional view of the SnSe-coated MF structure, and the inset shows the thickness of the deposited SnSe, which is approximately 190 nm. Additionally, the SnSe-coated MKR is put onto the MgF_2_ substrate, which provides stable support.

**Figure 3:**
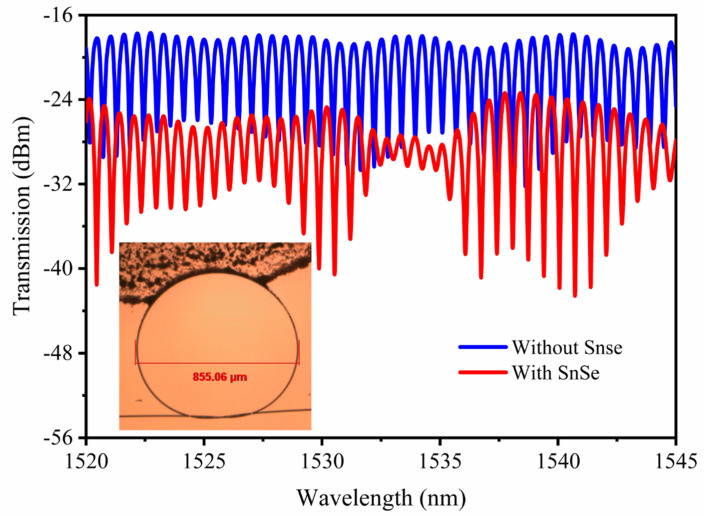
Transmission spectra of the MKR with and without SnSe nanosheets in the range from 1520 to 1545 nm. The blue line represents before deposition and the red line represents after deposition of SnSe nanosheets. The inset is the microscope image of the MKR overlaid with SnSe nanoplates. The diameter of MKR is 855.06 μm.

The micro-resonator is produced by making a knot in a piece of microfiber. The knot diameter is approximately 855.06 μm. The transmission is measured by connecting a tunable laser source (TLS, ANDO-AQ4321D) to one end of the MKR, whereas the other end is connected to an optical spectrum analyzer (OSA, YOKOGAWA-AQ6317C). Figure 3 shows the measured transmission spectrum of the MKR, from which we can deduce that it has a free spectral range (FSR) of approximately 0.65 nm, a Q-factor [23] of approximately 43,909, and an extinction ratio (ER) of approximately 17.2 dB at the resonance wavelength of approximately 1539.4 nm. Where the FSR can be expressed as the difference Δλ of two adjacent resonator wavelengths near λ_res_ (resonant wavelength), the ER represents the energy difference between the adjacent peaks and troughs in the micro-knot fiber resonance spectrum, the Q-factor represents energy loss during one cycle of light in the MKR, which can be valuated via the ratio between the resonant wavelength of the device and the full width at half maximum (FWHM): Q = λ_res_/FWHM (see the supplementary material, Figure S1). The SnSe-coated microfiber resonator is fabricated by depositing SnSe nanoplates in the MKR, as shown in the inset of Figure 3, the black section of which is the coating area. The SnSe coating area is away from the knot region, and the length of the coating area is about one-fifth of the MKR. The SnSe-coated MKR sample is fixed at a basin made by UV adhesive onto a MgF_2_ substrate. The deposition of SnSe resulted in a loss of approximately 9 dB, and the spectrum of resonator was changed slightly. The enveloped spectrum generated from the interference between the fundamental mode and excited higher order microfiber mode [24,25]. The interaction between the evanescent wave and SnSe nanosheets leads to a clearer envelope.

## 3. Principle of the All-Optical SnSe-Coated Microfiber Modulation

**Figure 4:**
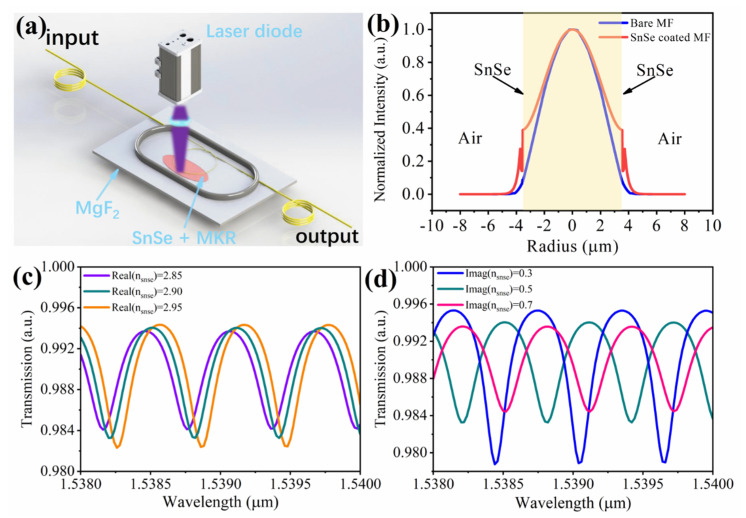
(**a**) Schematic diagram of the all-optical SnSe-coated microfiber modulator. (**b**) Mode field distribution of bare and SnSe-coated MF at 1550 nm. The yellow region represents the MF. (**c**) Simulated transmission spectrum of the SnSe-coated MKR changes with different real part of material refractive index. (**d**) Simulated transmission spectrum of the SnSe-coated MKR changes with different imaginary part of material refractive index.

Figure 4a shows the schematic diagram of the all-optical SnSe-coated microfiber modulator. When free-space illumination occurs by an external laser, the change in the holes and electrons will lead the SnSe nanosheets of the conductivity to change, and both the real and imaginary parts of the refractive index of the SnSe nanosheets are modulated, which further changes the mode effective refractive index of the SnSe-coated microfiber and finally tunes the ER and the wavelength shift of SnSe-coated MKR.

More specifically, the radial field distributions of bare and SnSe-coated MF along the white dashed line are plotted in Figure 4b. The simulation is implemented by the finite element method in COMSOL. In the model, a 190 nm SnSe layer is wrapped around the 7.1 μm MF. The calculation window was considered a rectangle with a size of 20 × 20 μm with a perfectly matched layer boundary condition. The wavelength is fixed at 1550 nm and the refractive indices of MF and SnSe nanosheet are 1.444 and 2.9 + 0.5i [26], respectively. Due to the index discontinuity, there is an abrupt variation at the interface between the MF and SnSe nanosheet. When changing the refractive index of the SnSe nanosheet, the field distributions and mode effective refractive index of the SnSe-coated microfiber are simultaneously changed.

For simplicity, the SnSe-coated MKR is considered the combination of the coupling region (two parallel bare microfibers) and the uniform optical path (SnSe-coated MF). The transmission spectrum of the SnSe-coated MKR is calculated using the following equation [27]:

(1) $$T=\frac{\exp\left(-\alpha L/2\right)\cdot \exp\left(j\beta L\right)-\kappa }{1-\exp\left(-\alpha L/2\right)\cdot \exp\left(j\beta L\right)\cdot \kappa }$$

where *α* and *β* are the loss and propagation constant of SnSe-coated microfiber, and *L* is the perimeter of MKR. Under the external laser radiation, the variation of the imaginary and real part of the SnSe material refractive index will change *α* and *β*. *κ* is the coupling radio that relates to the fractional coupling intensity at different ports of the MKR. Without loss of generality, the values of *κ* are fixed at 0.99. The extinction ratio (ER) variation is largely caused by *α* and the wavelength shift is caused by both *α* and *β*. As shown in Figure 4c, when decreasing imaginary part of the SnSe material refractive index, the ER is decreased and the spectrum shifts. Figure 4d shows the blue shift of the spectra caused by the decrease in the real part of the SnSe material refractive index.

## 4. Experimental Details, Results, and Discussion

In our experiment, an external pump light has been used for the all-optical modulation of SnSe-coated MKR. The external laser is placed approximately 10 cm above the sample and the focused spot is about 500 × 500 μm^2^ at the SnSe-coated microfiber. The wavelength of the illuminated laser is set as 405 nm. The transmission spectra of MKR without SnSe nanosheets are measured under the 405 nm illuminated laser.

**Figure 5:**
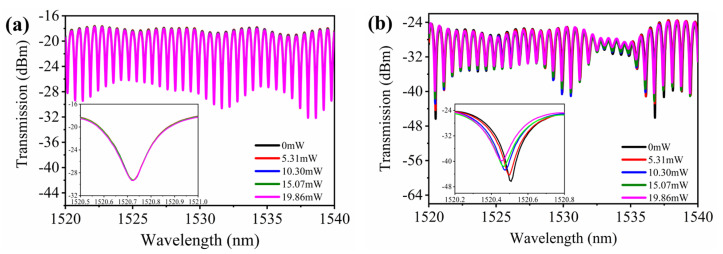
Transmission spectra of the MKR under the 405 nm illuminated laser. (**a**) Without SeSn nanosheets. (**b**) With SeSn nanosheets. Both insets are enlarged views of the resonance dips near 1520.5 nm.

As shown in Figure 5a, the transmission spectra do not vary with the power of the external pump. The transmission optical power loss is less than 0.4 dB when the external light power increased gradually with the gradient to a certain power. This indicates that the MKR sample owes the good stability to ensure the validity of the later all-optical modulated experiment. Figure 5b shows the transmission spectra of MKR with SnSe nanosheets. When illuminating by the 405 nm laser, the resonance wavelength shift increases with increasing external light power, and the ER decreases with increasing external light power. The inset clearly shows resonance dips at 1520.5 nm. For the external light power 19.86 mW, the resonance wavelength shift is 50 pm, and the ER is 15.6 dB.

**Figure 6:**
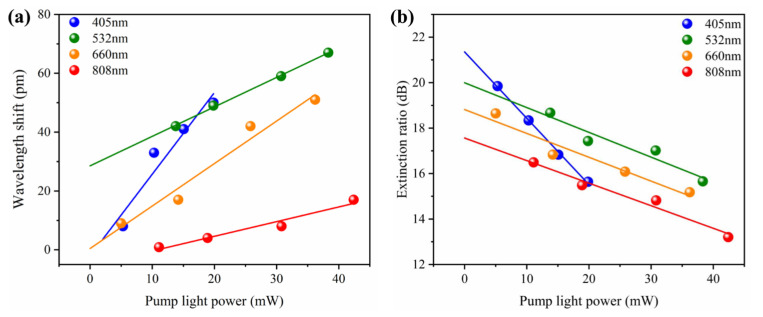
(**a**) Resonance wavelength shift at wavelength of 1520.5 nm versus the pump power of the violet, green, red and near-infrared light lasers, respectively. (**b**) ER at wavelength of 1520.5 nm versus the pump power of the four kinds of lasers, respectively.

To observe the broad tuning bandwidth of all-optical SnSe-coated MKR modulator, the violet (405 nm), green (532 nm), red (660 nm), and near-infrared (808 nm) laser are set as the external pump light, respectively. Transmission spectra of the MKR without SnSe or with SnSe at four lasers for external pump in Supplementary material Figures S2 and S3. The SnSe-based modulators can be implemented at wide spectral bands from ultraviolet to near-infrared due to the broadband photoresponse of SnSe nanosheets, as shown in Figure 1b. Figure 6a shows the resonance wavelength shift versus the different power of external violet/green/red/near-infrared light excitation. Solid balls in the figure represent the experimental data of various external pump lights, and the straight lines express the corresponding linear fitting. For all four external pump lights, the wavelength shift continually increases with increasing pump power. The tuning efficiencies of the wavelengths are 2.78, 1.00, 1.44, and 0.52 pm/mW for the violet, green, red, and near-infrared pump lights, respectively. As shown in Figure 6b, with an increase in the power of the external pump light, the ER of the device transmission spectra decreased gradually. The tuning efficiencies of ER are 0.29, 0.11, 0.10 and 0.10 dB/mW for the violet, green, red, and near-infrared pump lights, respectively. The ER tuning indicates that the amplitude of the transmission spectrum is tuned with light illumination. For both resonance wavelength shift and ER, the tuning efficiencies decrease with the increasing wavelength of the external pump light, which is consistent with the absorption spectrum of SeSn nanosheets (Figure 1b). The absorption of external pump-light energy by the SeSn nanosheets produced a carrier transition, which results in the change of the carrier concentration and distribution and further alters the refractive index real and imaginary parts of the SnSe nanosheets. The stronger the absorption coefficient, the greater the variation of the refractive index real and imaginary parts, and a larger tuning efficiency means that the all-optical SnSe-coated MKR modulator will have a better performance under the short-wavelength external pump light.

**Figure 7:**
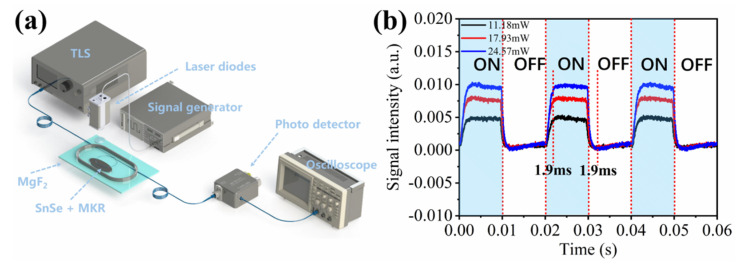
(**a**) Experimental setup for device response time measurement. (**b**) Response time of SnSe-coated MKR under 405 nm external pump light with various power levels.

To further characterize the device of the MKR with SnSe nanosheets, the response time is measured by the experimental setup shown in Figure 7a. The two ends of the sample are connected to the TLS (ANDO-AQ4321D) and the photodetector (Daheng Optics, DH-GDT-D020V). The signal generator (Tektronix, AFG3102) is used to control the optoelectronic cycle and power of the external pump light (405/532/660/808 nm), and the output signal of the photodetector is received by the oscilloscope (Siglent, SDS1102CFL). The signal wavelength of the TLS is 1536 nm, and the square waves of the signal generator are 20 ms and 50 Hz, respectively. All four types of external pump lights use three different power gradients. Figure 7b shows the the response-time waveforms of three cycles under 405 nm light exposures. The black, red, and blue waveforms represented different light powers. Response time of SnSe coated MKR under other lasers external pump light with various power in Supplementary material Figure S4. The rising and falling times under four different light irradiation conditions were all less than or equal to 2 ms, and three cycles intercepted under different pump-light powers show good repeatability. Additionally, there is no obvious relationship between the speed of response time and the power of the external pump light. An increase in power only enhanced the intensity of the response without changing the speed.

Based on the above results, an all-optical SnSe-coated MKR modulator is experimentally demonstrated with high tuning efficiency and a fast response time. In Table 1, the performances of various 2D material-based modulators are presented. Compared to other materials, the SnSe-based modulator has the general equilibrium ability for realizing the wide range of amplitude modulation, the high sensitivity and fast response speed. The SnSe-coated MKR modulator will satisfy the various application requirement in the all-optical information processing fields.

## 5. Conclusions

In conclusion, an SnSe-coated MKR coating is demonstrated for achieving the light-control-light functionality. In the preparation stage, the heat-flame taper-drawing method was used to fabricate microfiber and the liquid-phase exfoliation method for the SnSe nanoplates. Under 405 nm pump laser illumination, it has higher tuning efficiency for both ER (0.29 dB/mW) and wavelength shift (2.78 pm/mW) and exhibited a moderate averaged rise/fall time of less than 2 ms. The broad tuning bandwidth is confirmed by four external pump lights ranging from ultraviolet to near-infrared. The SnSe-coated MKR modulator has the advantages of broad tuning bandwidth, high tuning efficiency, compact size, easy fabrication, and low cost. Therefore, it is a great candidate for developing fiber-compatible all-optical modulators with other functionalities.

## Figures and Tables

**Table 1:** Units for magnetic properties.

2D Materials	ER (dB)	Tuning Efficiency of ER (dB/mW)	Tuning Efficiency of Wavelength Shift (pm/mW)	Response Time (ms)
Graphene [28]	1.5	0.02	1.29	-
Phosphorene [29]	17	-	4.0	2.5
WS_2_ [16]	15.3	0.4	0.84	120
In_2_Se_3_ [30]	-	0.815	-	1.6
SnS_2_ [31]	26	0.22	-	3.2
SnSe (this paper)	19.8	0.29	2.78	1.9

## Data Availability

The data presented in this study are available on request from the corresponding author.

## Supplementary Materials

The following supporting information can be downloaded at: https://www.mdpi.com/article/10.3390/nano12040694/s1, Figure S1: Transmission spectrum of micro-knot resonator; Figure S2: (**a**) Transmission spectra of the MKR without SnSe at different 405 nm laser powers for external pump. (**b**) Transmission spectra of the MKR without SnSe at different 532 nm laser powers for external pump. (**c**) Transmission spectra of the MKR without SnSe at different 660 nm laser powers for external pump. (**d**) Transmission spectra of the MKR without SnSe at different 808 nm laser powers for external pump; Figure S3: (**a**) Transmission spectra of the MKR coated with SnSe at different 405 nm laser powers for external pump. (**b**) Transmission spectra of the MKR coated with SnSe at different 532 nm laser powers for external pump. (**c**) Transmission spectra of the MKR coated with SnSe at different 660 nm laser powers for external pump. (**d**) Transmission spectra of the MKR coated with SnSe at different 808 nm laser powers for external pump; Figure S4: (**a**) Response time of SnSe coated MKR under 405 nm external pump light with various power. (**b**) Response time of SnSe coated MKR under 532 nm external pump light with various power. (**c**) Response time of SnSe coated MKR under 660 nm external pump light with various power. (**d**) Response time of SnSe coated MKR under 808 nm external pump light with various power.
