# Supplementary material for: SnSe-Coated Microfiber Resonator for All-Optical Modulation
Source: Nanomaterials (Basel). 2022 Feb 19;12(4):694. doi: 10.3390/nano12040694 (PMC8880113; doi:10.3390/nano12040694)
Supplement: Supplementary file 1 [file nanomaterials-12-00694-s001.zip › nanomaterials-1572276-supplementary.pdf]

nanomaterials

Supplementary material:

SnSe-coated Microfiber Resonator for All-optical Modulation

Lei Chen [1,†], Jingyuan Ming [1,†], Zhishen Zhang [2], Jumei Shang [1], Lingyun Yu [1], Heyuan Guan [3,*], Weina Zhang [1], Zefeng Xu [1], Wentao Qiu [3,*], Zhe Chen [3] and Huihui Lu [3]

[1] Guangdong Provincial Key Laboratory of Optical Fiber Sensing and Communications, Department of Optoelectronic Engineering, Jinan University, Guangzhou 510632, China; lynnchan538@stu2018.jnu.edu.cn (L.C.); mingjingyuan@outlook.com (J.M.); 1934021043@stu2019.jnu.edu.cn (J.S.); llingyunyu@163.com (L.Y.); vina_zhang001218@163.com (W.Z.); xuzefeng134210@163.com (Z.X.)

[2] School of Physics and Optoelectronic Technology, South China University of Technology, Guangzhou 510641, China; zhishenzhang@yeah.net (Z.Z.)

[3] Key Laboratory of Optoelectronic Information and Sensing Technologies of Guangdong Higher Education Institutes, Department of Optoelectronic Engineering, Jinan University, Guangzhou 510632, China; thzhechen@163.com (Z.C.); thuihuilu@jnu.edu.cn (H.L.)

* Correspondence: ttguanheyuan@jnu.edu.cn (H.G.); qiuwentao@jnu.edu.cn (W.Q.)

† L.C. and J.M. contributed equally to this work.

I. Explanation of FSR, ER and Q-factor.

1. The free spectral range (FSR) represents the distance between two adjacent wavelengths in the resonant spectrum of the ring cavity. The FSR determines the working bandwidth of the device. The larger the FSR, the wider the working bandwidth of the device. It is related to the diameter of the micro-knot fiber. FSR can be expressed as the difference $\Delta\lambda$ of two adjacent resonator wavelengths near λ_{res}(resonant wavelength):

$$FSR \approx \Delta\lambda \approx \frac{\lambda^2}{4\pi n_{eff} L}$$

Where n_{eff} is the effective refractive index of micro-knot fiber, L is the circumference of the micro-knot fiber.

Experimentally, it can be estimated from the transmission spectrum, like Figure R1.

2. The full width at half maximum (FWHM) is also called the half width of the resonance peak or the 3 dB bandwidth. It represents the spectral wavelength width when the response amplitude of the device drops to half of the peak value.

3. The extinction ratio(ER) represents the energy difference between the adjacent peaks and troughs in the micro-knot fiber resonance spectrum, it is shown in Figure R1. It is mainly related to the coupling efficiency of the microfiber ring cavity. The higher the coupling efficiency, the higher the extinction ratio of the microfiber ring, and the Q-factor.

4. The Q-factor represents energy loss during one cycle of light in the MKR which can be valuated via the ratio between the resonant wavelength of the device and the 3 dB bandwidth. It reflects the steepness of the resonant wavelength of the micro-knot resonator at a certain location.

$$Q = \lambda_{res} / FWHM$$

It is related to the coupling loss of the device. If the Q value is larger, the light will stay in the ring longer and the wavelength selectivity will be better.

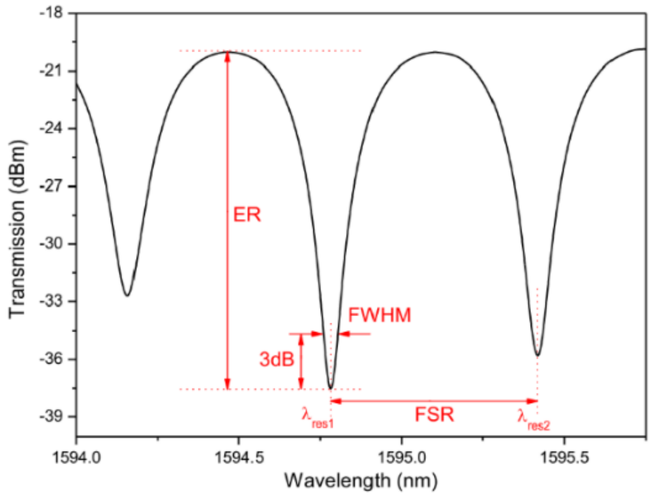

Figure S1. Transmission spectrum of micro-knot resonator.

Some explanation of these parameters from the reference [24].

II. We show the response time, the transmission spectra of the MKR without SnSe and with SnSe for four lasers as below:

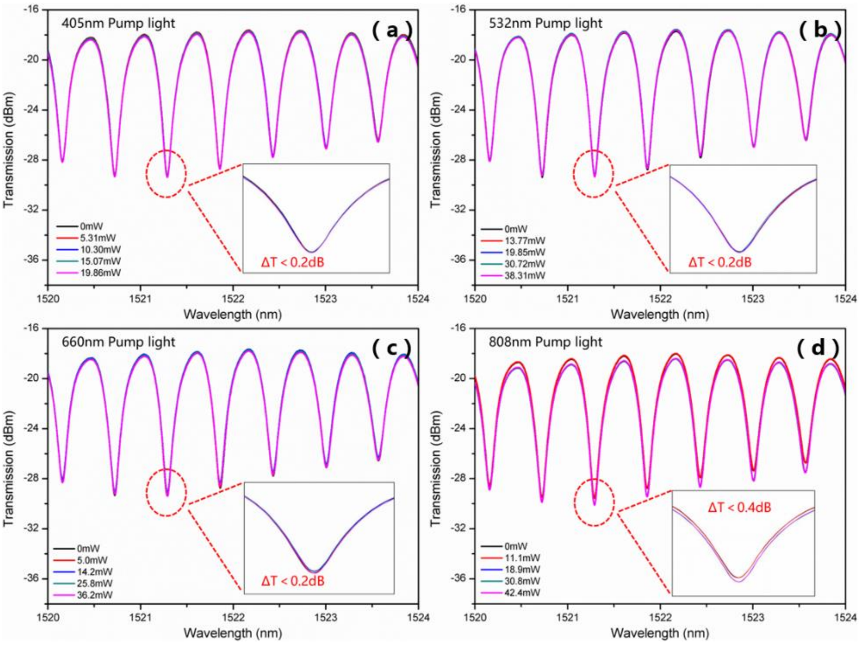

Figure S2. (**a**) Transmission spectra of the MKR without SnSe at different 405 nm laser powers for external pump. (**b**) Transmission spectra of the MKR without SnSe at different 532 nm laser powers for external pump. (**c**) Transmission spectra of the MKR without SnSe at different 660 nm laser powers for external pump. (**d**) Transmission spectra of the MKR without SnSe at different 808 nm laser powers for external pump.

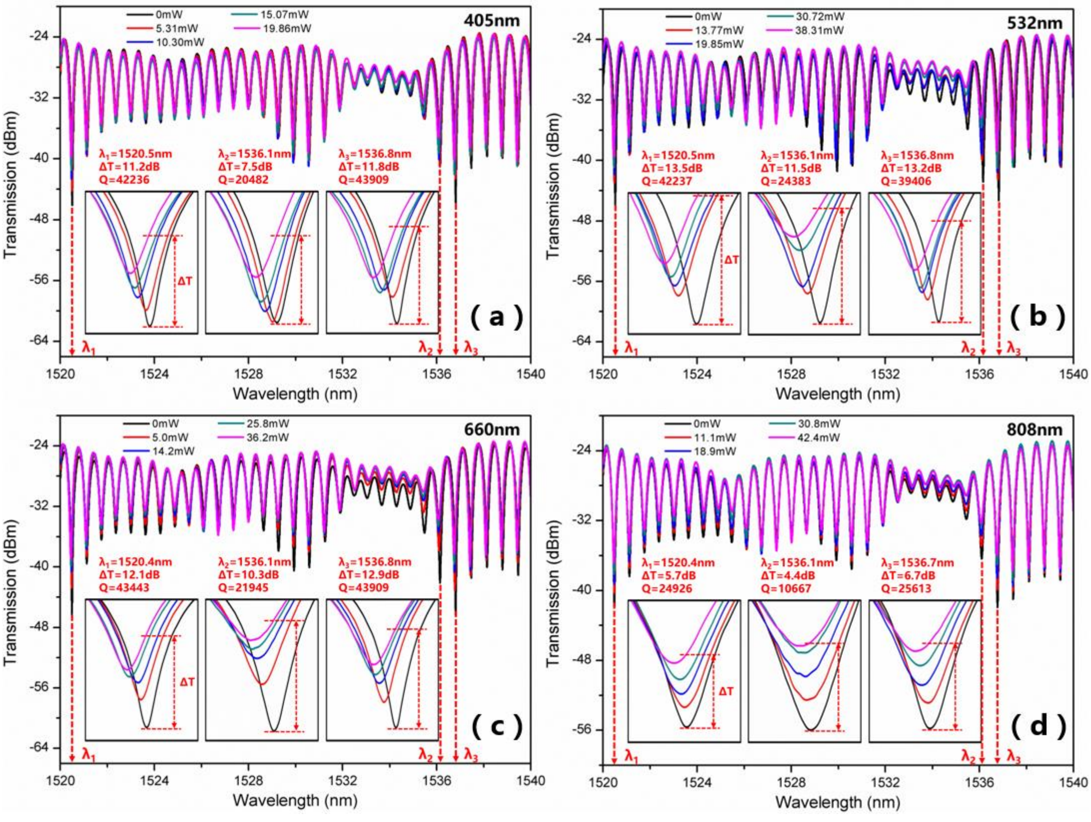

Figure S3. (**a**) Transmission spectra of the MKR coated with SnSe at different 405 nm laser powers for external pump. (**b**) Transmission spectra of the MKR coated with SnSe at different 532 nm laser powers for external pump. (**c**) Transmission spectra of the MKR coated with SnSe at different 660

nm laser powers for external pump. (**d**) Transmission spectra of the MKR coated with SnSe at different 808 nm laser powers for external pump.

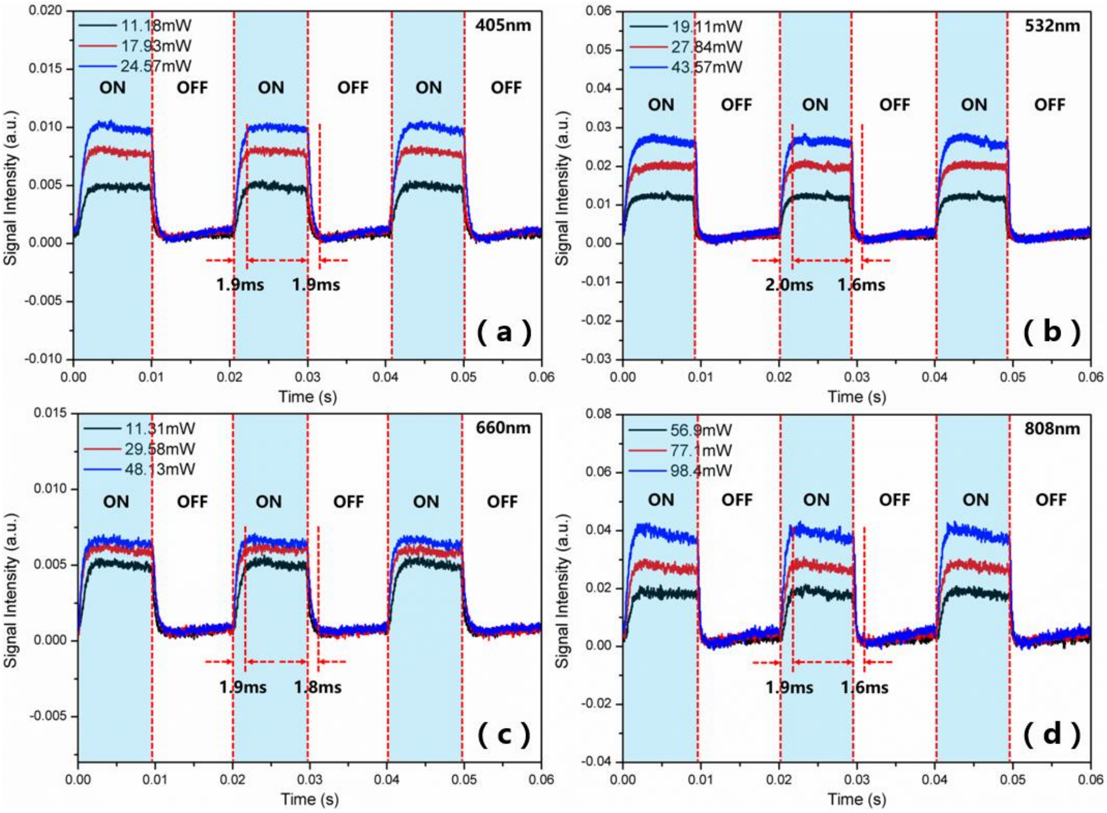

Figure S4. (**a**) Response time of SnSe coated MKR under 405 nm external pump light with various power. (**b**) Response time of SnSe coated MKR under 532 nm external pump light with various power. (**c**) Response time of SnSe coated MKR under 660 nm external pump light with various power. (**d**) Response time of SnSe coated MKR under 808 nm external pump light with various power.
